# Higher interest to continue COVID-19 practice recommendations in non-pandemic times among German GPs with better crisis leadership skills (egePan study)

**DOI:** 10.1186/s12913-024-11855-7

**Published:** 2024-11-13

**Authors:** Benjamin Aretz, Yelda Krumpholtz, Simon Kugai, Nicola Amarell, Manuela Schmidt, Birgitta Weltermann

**Affiliations:** 1 Institute of General Practice and Family Medicine, University Hospital Bonn, University of Bonn, Bonn, Germany; 2 Institute of Political Science and Sociology, University of Bonn, Bonn, Germany

**Keywords:** Pandemic preparedness, General practice changes, COVID-19, Nationwide survey, General practitioners, Primary care

## Abstract

**Background:**

The German College of General Practitioners and Family Physicians (DEGAM) issued a COVID-19 guideline with eleven recommendations to support primary care services during the pandemic. Their use in general practices beyond the pandemic can contribute to pandemic preparedness. This study analysed general practitioners’ (GPs) interest in applying recommended organisational changes in non-pandemic times.

**Methods:**

Data from the German egePan GP survey (*n* = 516 GPs) - a multi-level clustered randomised web-based survey - were analysed. GPs’ interest in the future application of the eleven guideline recommendations was calculated. In addition, each recommendation was evaluated by the GPs using a Net-Promoter-Score (NPS range − 100 to 100). A linear regression model identified GP and practice characteristics associated with a higher interest in applying recommendations in non-pandemic times.

**Results:**

98.5% of the GPs indicated the intention to implement at least one guideline recommendation prospectively: disinfectant dispensers at the entrance (86%), optimised consultation scheduling to reduce waiting times (83%), and glass screens in the reception area (72%), which also received the highest NPS scores. In contrast, lower interest was observed for items such as insurance card readers handled by patients (48%), only selected staff treating infectious patients (44%), and video consultations for patients with infections (26%). A higher interest to implement recommendations in non-pandemic times was associated with a higher crisis leadership score (*p* < 0.001), using the Corona-Warn-App (*p* = 0.007), and being a female GP (*p* = 0.045). In addition, GPs from Western, Northern, and Southern, and those with a higher patient volume per three months, were more interested in future implementation.

**Conclusions:**

Overall, GPs demonstrated the readiness to follow the DEGAM COVID-19 guideline outside pandemic periods, establishing them as key contributors to pandemic preparedness in Germany.

**Supplementary Information:**

The online version contains supplementary material available at 10.1186/s12913-024-11855-7.

## Background

The COVID-19 pandemic was a public health challenge, that required fundamental adoptions from the healthcare system worldwide [2, 4, 12, 37]. General practitioners (GPs) played a vital role during the pandemic, as they often served as the initial point of contact and assumed responsibility for patients’ short-term and long-term care [15, 18, 35, 38]. Moreover, GPs treated the vast majority of suspected and confirmed ambulatory COVID patients (> 90%) in Germany [13].

In the pandemic, GP practices faced unparalleled organisational challenges and constraints when delivering high-quality care [5, 14, 33]. These challenges included the limited availability of resources regarding staff, inadequate infrastructure, and, particularly in the initial phases of the pandemic, a shortage of personal protective equipment [20, 21]. A key challenge for all primary care institutions was to create a set-up that protects the primary care workforce, while allowing safe and efficient diagnostic and therapeutic care for patients with and without (suspected) COVID-19 [19, 39].

By their high responsibility to the COVID-19 pandemic, primary care facilities worldwide changed their routines to provide safe services, sustain health care, and avoid a systemic overload [15, 19, 30]. Guidelines from various professional boards supported these changes. In Germany, the College of General Practitioners and Family Physicians (DEGAM) recommended various practice changes addressing structural and procedural aspects of daily care, e.g., separate consultation rooms and consultation times for patients with and without an infection, distance markers in the reception area, and the provision of hand disinfectant via dispensers at the practice entrance [3]. However, there were significant variations in the guidelines across different countries worldwide: A systematic review of 138 guidelines worldwide revealed that 85 (= 62%) contained at least one statement that differed from another guideline, resulting in a total of 223 diverging statements and 66 specific clusters of guidelines [27].

The World Health Organization defines pandemic preparedness as a ‘continuous process of planning, exercising, revising and translating into action national and sub-national pandemic preparedness’ [40]. To ensure future (regional) pandemic preparedness, country-specific analyses of the future relevance of the COVID guidelines in non-pandemic times are needed. GPs who face these guidelines in their daily practice play a vital role in assessing the relevance of COVID-19 recommendations, even regarding action plans for future pandemic preparedness.

Using a web-based survey, this German study analysed GPs’ interest in applying pandemic-driven organisational changes recommended in the DEGAM COVID-19 guideline, also in non-pandemic times, and to identify GP and GP practice characteristics contributing to its future application.

## Materials and methods

### Research consortium, financing, and ethics statement

This research was conducted as part of the egePan Unimed project, a nationwide research consortium by the Network of University Medicine (NUM). The project’s primary objective was developing, testing, and implementing evidence-based adaptive care structures and processes for pandemic management. The German Federal Ministry of Education and Research (BMBF) funded this project under grant number 01KX2021. The study was approved by the Ethics Committee of the Medical Faculty of the University of Bonn (reference number: 419/20, date of approval: 5 February 2021).

### Sampling

This study used the egePan GP survey data based on a multi-level clustered randomised sample of GPs, chosen from the entire population of working GPs in Germany with a valid email address. The list was obtained from ArztData AG, a specialised provider of physician addresses. The sampling process involved two steps.

First, the data were divided into clusters based on federal states and regional population density. Within the 16 German states, 64 county layers were randomly selected. Second, each cluster was further stratified into four layers based on practice type and nature of employment: ‘GP in own practice’, ‘employed in practice’, ‘leader of an ambulatory healthcare centre’, and ‘employed in an ambulatory healthcare centre’. From each layer, 30% of GPs were randomly chosen and invited to participate in our web-based survey. Survey participation was anonymous to ensure data privacy.

### Questionnaire and used instruments

We developed a survey to gather insights into regional pandemic management, focusing on successful and unsuccessful aspects. Frequent themes included communication strategies, collaborative efforts, testing capabilities, hygiene protocols, personal protective equipment, and personnel shortages. The items of the web-based questionnaire were based on a systematic literature review on pandemic preparedness and the DEGAM COVID-19 guideline. The questionnaire was piloted with 55 participants using the tool www.unipark.com, which adheres to German data protection laws. The egePan project encompassed various work packages involving numerous university hospitals throughout Germany. Participating institutes and clinics represented diverse specialities, including internal medicine, virology, microbiology, hygiene, anesthesiology, public health, psychiatry, and nursing. All participants for the pilot study held leadership or directorial positions within their respective institutes or clinics, bringing expertise in their fields and contributing significantly to the broader egePan project initiative.

Based on the insights gained during the pilot phase, the questionnaire was revised and finalised for the main survey. The final questionnaire [1] comprised two main parts. The first part focused on the socio-demographic characteristics of the GPs and their practices: gender, practice experience, practice type, practice size, use of the German COVID tracing app (Corona-Warn-App), and the vaccination status of the practice staff.

The second part of the questionnaire referred, among others, to the GPs’ perspectives in dealing with COVID-19 and their interest in applying the following eleven organisational practice changes proposed by the DEGAM also in non-pandemic times:

1. *Disinfectant dispensers at the entrance*.
2. *Optimised consultation scheduling to reduce waiting times*.
3. *Glass screens in the reception area*.
4. *Separate consultations for infectious patients*.
5. *Face masks are obligatory for patients with respiratory infections*.
6. *Testing for COVID only if personal protective equipment is sufficient*.
7. *Phone consultations for patients with respiratory infections*.
8. *Prescriptions by mail*.
9. *Insurance card reader handled by patients*.
10. *Only selected staff treat infectious patients*.
11. *Video consultations for patients with infections.*

GPs were asked to rate their likelihood for future implementation by themselves using the following question: *‘How likely is it that you will use the following protective measures even in non-pandemic times?’* (response options: 10-point Likert scale with higher values meaning better ratings). Following the concept of the Net Promoter Score (detailed information is provided in the section on statistical analysis), a score from 1 to 7 was assumed as a negative rating, a score of 8 and 9 described a neutral value, and a score greater than 9 was classified as a positive evaluation.

A total of 10,600 invitations were emailed. After the cleansing of invalid email addresses, 9,287 GPs remained. The survey was open from 17 March 2021 until 17 June 2021, and a reminder was sent after four weeks to enhance the response rate.

### Crisis leadership skills

Crisis leadership skills were measured by the C-LEAD scale. The C-LEAD scale comprises nine items evaluating self-perceived crisis leadership competencies: efficacy as a leader, learning goal orientation, intelligence, divergent thinking, practising crisis response protocols, procedural crisis preparedness, motivation to lead in a crisis, crisis leader role-taking, and crisis decision-making. These items are rated on a 7-point scale ranging from 1 (strongly disagree) to 7 (strongly agree). The original questionnaire of the egePan survey is published [1]. The individual item scores were summed up to calculate the overall C-LEAD score, ranging from 9 to 63 [11].

### Socio-demographic variables

The independent variables used in this study encompassed the following factors: gender (female, male), years of experience as a GP, practice size categorised into quartiles based on the number of employees/ co-workers, the proportion of employees who tested positive for COVID-19 in percent, quartiles of the number of employees in the practices, the geographical sub-region of the GP’s location in Germany (Northern Germany: Schleswig-Holstein, Hamburg, Bremen, Lower Saxony; Southern Germany: Bavaria, Baden-Württemberg; Eastern Germany: Thuringia, Saxony, Saxony-Anhalt, Berlin, Brandenburg, Mecklenburg Western Pomerania; Western Germany: North-Rhine-Westphalia, Rhineland Palatinate, Saarland), patient load per quarter (up to 1000, 1001–1500, 1501–2000, 2001 or more), usage of the Corona-Warn-App (yes, no), perceived workload (ranging from very low to very high divided into five levels), and crisis leadership competencies measured using the German version of the validated Crisis Leader Efficacy in Assessing and Deciding scale (C-LEAD) [16].

### Statistical analysis

First, absolute and relative frequencies, as well as the mean and standard deviation for metric variables, were calculated to describe the distribution of GP and practice characteristics within the study population.

Second, we evaluated the GPs’ interest in applying the organisational changes in non-pandemic times in the total sample, and separately for GPs from Western, Northern, Eastern, and Southern Germany. Values equal to or larger than 6 were signed as a positive interest to apply the organisational changes in non-pandemic times, because the variables used a 10-point Likert scale ranging from 1 to 11. Then, the percentages of GPs’ positive interest for each of the eleven organisational practice changes proposed by the DEGAM were calculated.

Third, the Net Promoter Score (NPS) for each of the eleven organisational practice changes was computed. The concept of NPS stems from marketing research and indicates the degree of recommendation of a product to others. NPS is an aggregated measure reflecting the extent of satisfaction among GPs with the implemented organisational practice changes ranging from − 100 to 100. NPS was determined by subtracting the aggregated relative frequency of detractors (those who rated between 1 and 6, denoted as ($\:\frac{{f}_{D}}{n}$)) from the relative frequency of promoters (those who rated between 9 and 11, denoted as ($\:\frac{{f}_{P}}{n}$)):

1 $$\:NPS=\:\left(\frac{f_P}n\right)-\left(\frac{f_D}n\right)$$

Values lower than 0 indicate a bad rating, values greater than 0 indicate a good rating, values greater than 20 indicate a very good rating, values greater than 50 indicate an excellent rating, and values greater than 80 indicate an outstanding rating [29].

The differences in the average NPS between the German subregions were tested using analysis of variance (ANOVA).

Fourth, a linear OLS (ordinary least squares) regression model (ii) was estimated to explore the role of crisis leadership skills and other essential determinants for the GPs’ overall interest in applying the organisational changes in their practices also in non-pandemic times:

2 $$\:{y}_{i}={\beta\:}_{0}+{\beta\:}_{1}{X}_{i}+\dots\:+{\beta\:}_{n}+{\epsilon_{i}}$$

For this purpose, the ratings for the eleven organisational changes were summarised into a composite score ranging from 0 to 100 (with higher values indicating a more positive intention).

Robust standard errors accounted for heteroscedasticity, which was studied using the Breusch-Pagan test (*p* < 0.001). Multicollinearity did not bias the estimations (VIF = 1.93 < 5). A significance level of 0.05 was employed. The statistical analysis was conducted using STATA 17 [34].

## Results

### Characteristics of the participating GPs

Of the 9,287 invited GPs, 516 GPs completed the questionnaire and provided valid responses regarding their interest in applying the DEGAM COVID-19 guideline, translating to a net response rate of 5.56%.

Notably, the survey coincided with the start of the nationwide vaccination campaign, which heavily involved GPs who worked in their practices and public vaccination centres. However, the survey could not be postponed due to funding issues. Of the GPs, 35.9% came from the Northern, 15.9% from the Southern, 20.9% from the Eastern, and 27.3% from the Western German region (Table 1). On average, GPs had a practice experience of 18.44 years (SD = 9.55) and perceived the workload during the COVID-19 pandemic as high (mean: 4.13 on a five-point scale). Furthermore, GPs were characterised by positive crisis leadership qualities as indicated by the C-LEAD scores (mean: 47.13, min = 10, max = 63). GP practice owners employed, on average, 7.69 (SD = 7.78) practice assistants and/or employed GPs. As of June 2021, approximately 11% of the practice personnel had tested positive for COVID-19.

**Table 1:** GP and GP practice characteristics (*n* = 516)

	Total sample (*n* = 516)	
	n	%
**Gender**		
Male	304	58.9
Female	212	41.1
**Years as a GP**		
1. Quartile (0–11 years)	143	27.7
2. Quartile (12–18 years)	125	24.2
3. Quartile (19–27 years)	129	25.0
4. Quartile (28 years or more)	119	23.1
**Number of employees/ co-workers**		
1. Quartile (0–4 employees)	168	32.6
2. Quartile (5–6 employees)	121	23.5
3. Quartile (7–9 employees)	98	19.0
4. Quartile (10 or more employees)	129	25.0
**Ratio employees tested positive for COVID/ number of employees**, mean (SD)	0.11 (0.23)	
**Region**		
Northern Germany	185	35.9
Southern Germany	82	15.9
Eastern Germany	108	20.9
Western Germany	141	27.3
**Patients per quarterly period**		
Up to 1000	94	18.2
1001–1500	162	31.4
1501–2000	92	17.8
More than 2000	168	32.6
**COVID-app use (Corona-Warn-App)**		
Yes	296	57.4
No	220	42.6
**Workload**		
(Very) low	20	3.9
Medium	83	16.1
(Very) heavy	413	80.0
**Crisis leadership score (C-LEAD)**, mean (SD)	47.13 (8.51)	

### The interest in applying the DEGAM-recommended COVID-19 guideline in non-pandemic times

The vast majority (98.45%, *n* = 508) reported that they would incorporate at least one recommendation from the DEGAM COVID-19 guideline into their practices in non-pandemic times. In contrast, only 5.43% (*n* = 28) planned to adopt all recommendations. Interest was exceptionally high for ‘disinfectant dispensers at the entrance’ (86.25%), optimised consultation scheduling’ (82.95%), ‘installing glass screens in the reception area’ (72.09%), ‘phone consultations for patients with respiratory infections’ (71.90%), and ‘face masks obligatory for patients with respiratory infections’ (70.74%) (Table 2). The average ratings for the single recommendations confirm these findings (Supplementary Table S1). Interestingly, there were significant variations in the interest to apply the recommendations between different German subregions for

**Table 2:** GPs interest in applying organisational changes in non-pandemic times: proportions of positive interest in %

	Regional level					ANOVA
**Structure/Process**	Total sample (*n* = 516)	GPs from Western Germany (*n* = 185)	GPs from Northern Germany (*n* = 82)	GPs from Eastern Germany (*n* = 108)	GPs from Southern Germany (*n* = 141)	F-statistics (p-value)
Disinfectant dispensers at the entrance	86.05	90.81	86.59	74.07	88.65	**5.89** **(< 0.001)**
Optimised consultation scheduling to reduce waiting times	82.95	85.95	82.93	72.22	87.23	**3.99** **(0.008)**
Glass screens in the reception area	72.09	73.51	73.17	67.59	73.05	0.46 (0.711)
Separate consultations for infectious patients	69.38	65.95	78.05	64.81	72.34	1.86 (0.135)
Face masks are obligatory for patients with respiratory infections	70.74	67.57	75.61	67.59	74.47	1.10 (0.349)
Testing for COVID only if personal protective equipment is sufficient	62.40	61.62	65.85	61.11	62.41	0.18 (0.911)
Phone consultations for patients with respiratory infections	71.90	73.51	69.51	64.81	76.60	1.57 (0.197)
Prescriptions by mail	60.47	64.86	50.00	59.26	61.70	1.81 (0.145)
Insurance card reader handled by patients	48.06	45.41	54.88	29.63	61.70	**9.52** **(< 0.001)**
Only selected staff treat infectious patients	43.80	41.08	43.90	40.74	49.65	0.97 (0.405)
Video consultations for patients with infections	25.58	27.57	19.51	21.30	29.79	1.44 (0.230)

- Disinfectant dispensers at the entrance (*p* < 0.001),
- Optimised consultation scheduling to reduce waiting times (*p* = 0.008),
- Insurance card reader handled by patients (*p* < 0.001),

with a lower interest among GPs from Eastern Germany.

### The net promoter scores (NPS)

Overall, the NPS approach revealed positive ratings regarding the future use of seven of the eleven guideline recommendations (Table 3).

**Table 3:** Net-promoter-scores of the eleven guidelines recommendations as rated by German GPs: total and by region

	Regional level				
**Structure/Process**	Total sample (*n* = 516)	GPs from Western Germany (*n* = 185)	GPs from Northern Germany (*n* = 82)	GPs from Eastern Germany (*n* = 108)	GPs from Southern Germany (*n* = 141)
Disinfectant dispensers at the entrance	64.8	76.7	67.2	34.8	69.4
Optimised consultation scheduling to reduce waiting times	44.3	57.7	33.3	20.5	51.4
Glass screens at reception	22.4	29.7	27.0	16.7	14.8
Separate consultations for infectious patients	9.9	7.6	14.8	8.5	11.5
Face masks are obligatory for patients with respiratory infections	9.6	7.1	16.7	4.8	12.6
Phone consultations for patients with respiratory infections	4.5	9.0	-9.7	-8.2	19.2
Testing for COVID only if personal protective equipment is sufficient	2.7	-1.3	8.3	-5.5	10.4
Prescriptions by mail	-15.4	-5.7	-34.3	-8.2	-21.7
Insurance card reader handled by patients	-20.1	-23.8	-6.8	-54.0	4.9
Only selected staff treat infectious patients	-42.3	-47.8	-38.0	-46.7	-33.9
Video consultations for patients with infections	-72.5	-68.3	-81.3	-75.5	-70.3

Excellent ratings were given for the following three recommendations:

- Glass screens at the reception (NPS = 22.4),
- Optimised consultation scheduling to reduce waiting times (NPS = 44.3),
- Disinfectant dispensers at the entrance (NPS = 64.8).

Positive ratings were noted for the following four recommendations:

- Separate consultations for infectious patients (NPS = 9.9),
- Face masks obligatory for patients with respiratory infections (NPS = 9.6),
- Phone consultations for patients with respiratory infections (NPS = 4.5),
- Testing for COVID only if personal protective equipment is sufficient (NPS = 2.7).

In contrast, negative ratings were given for the following three organisational recommendations:

- Prescriptions by mail (NPS = -15.4),
- Insurance card reader handled by patients (NPS = -20.1),
- Only selected staff treats infectious patients (NPS = -42.3),
- Video consultations for patients with infections (NPS = -72.5).

### Associations between GP characteristics and interest in implementing guidelines recommendations in non-pandemic times

**Fig. 1:**
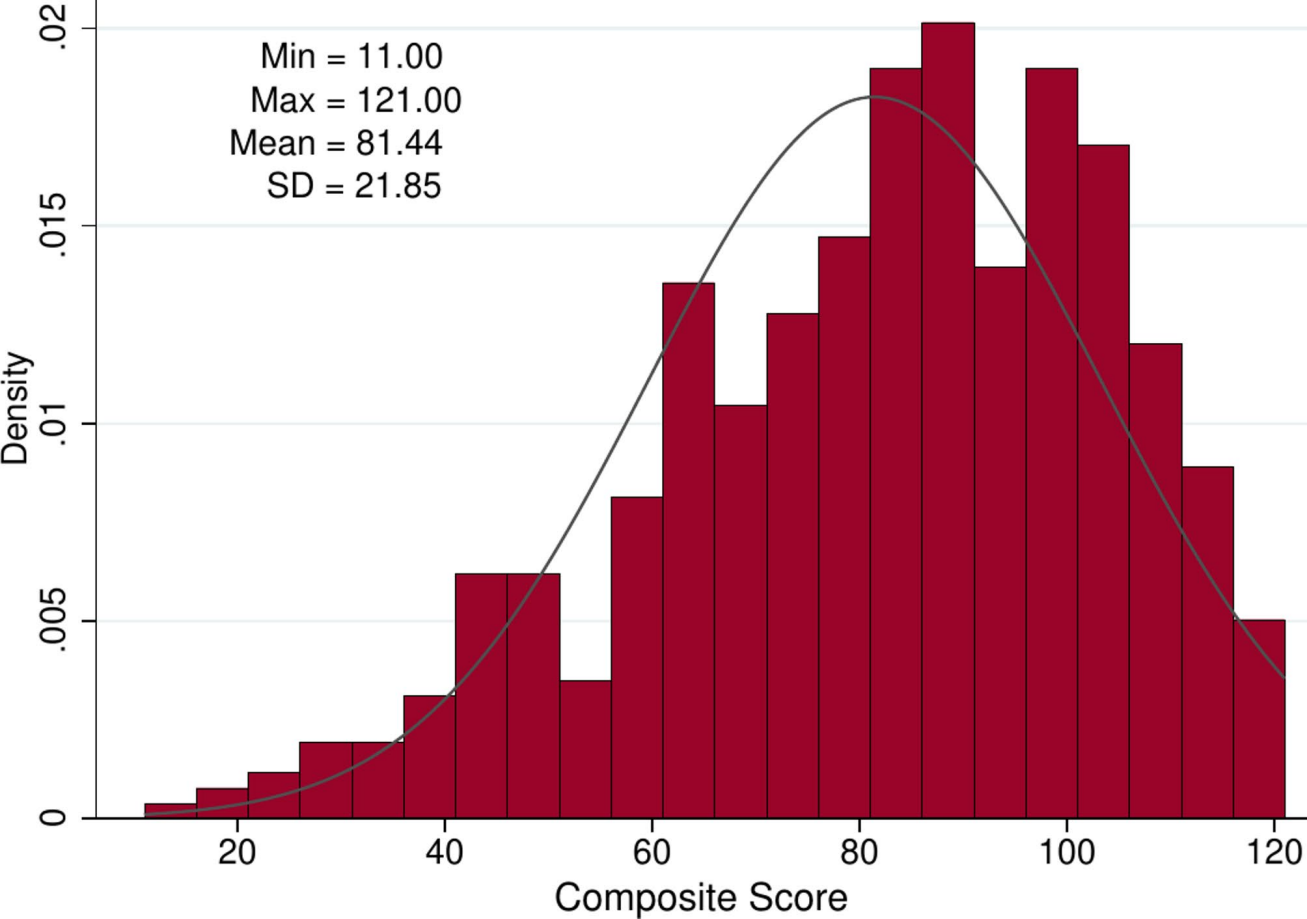
Overall GPs’ interest to continue the DEGAM-recommended COVID-19 recommendations in non-pandemic times

Overall, GPs were highly interested in applying the organisational changes in non-pandemic times (Fig. 1). The linear regression model (Table 4) revealed several factors associated with a higher interest in implementing recommendations in non-pandemic times: a higher crisis leadership score (*p* < 0.001), using the Corona-Warn-App (*p* = 0.007), and being a female GP (*p* = 0.045).

**Table 4:** GP and GP practice characteristics associated with the interest in applying the DEGAM COVID-19 guideline in non-pandemic times (*n* = 516)

Variable	Regression coefficient β	95%-CI	*p*-value
**Gender**			
Male	**-3.81**	-7.54, -0.08	0.045
Female	ref.		
**Years as a GP (practice experience)**			
1. Quartile (0–11 years)	ref.		
2. Quartile (12–18 years)	0.13	-4.49, 4.75	0.957
3. Quartile (19–27 years)	-3.33	-8.52, 1.87	0.209
4. Quartile (28 years or more)	**-6.08**	-6.08, 2.77	0.028
**Number of employees/ co-workers**			
1. Quartile (0–4 employees)	ref.		
2. Quartile (5–6 employees)	1.61	-3.70, 6.91	0.552
3. Quartile (7–9 employees)	3.85	-2.24, 9.94	0.215
4. Quartile (10 or more employees)	1.36	-4.89, 7.60	0.670
**Ratio employees tested positive for COVID/ number of employees**	0.69	-7.40, 8.77	0.868
**Region**			
North	-1.60	-7.40, 4.20	0.545
South	ref.		
East	**-6.08**	-11.58, -0.57	0.031
West	-1.60	-7.40, 4.20	0.588
**Patients per quarterly period**			
Up to 1000	ref.		
1001–1500	**6.05**	0.23, 11.88	0.042
1501–2000	**9.63**	3.31, 15.95	0.003
2001 and more	**8.95**	2.38, 15.53	0.008
**COVID-app (Corona-Warn-App)**			
Yes	ref.		
No	**-5.03**	-8.71, -1.36	0.007
**Workload**			
(Very) low	ref.		
Medium	1.04	-8.49, 10.56	0.831
(Very) heavy	7.19	-1.31, 15.69	0.097
**Crisis leadership score**, mean (SD)	**0.41**	0.19, 0.64	< 0.001

*Bold point estimators denote significant results with *p* < 0.05

Also, higher patient volume per three months (> 1.000 patients per quarter) increased GPs’ interest in implementation. In contrast, having 28 or more years of experience as a GP (β = -6.08, *p* = 0.028), practising in Eastern Germany (β = -6.08, *p* = 0.031), and not using the Corona-Warn-App (β = -5.03, *p* = 0.007) was associated with less interest in the future application of recommendations.

In addition, a noteworthy interaction between GPs’ crisis leadership skills and their location of residence was identified (*p* < 0.001). The models were thus segmented based on the German subregions, which showed that crisis leadership skills (C-LEAD) were positively associated with the GPs’ interest in applying the organisational changes in non-pandemic times in Western (β = 0.45, *p* = 0.021, 95% CI: 0.07, 0.83), Northern (β = 0.95, *p* = 0.008, 95% CI: 0.25, 1.65), and Eastern Germany (β = 0.74, *p* = 0.002, 95% CI: 0.29, 1.19), but not in Southern Germany.

## Discussion

### Summary of the findings

The DEGAM COVID-19 guideline was highly accepted even in non-pandemic times among German GPs: more than 98% intended to apply at least one recommendation beyond the pandemic. Seven of the eleven organisational practice changes received good or excellent ratings concerning future implementation, with a higher interest among physicians with a higher crisis leadership score, female gender, using the Corona-Warn-App, and those with a higher patient volume in three months. The observed regional difference with less interest in the Eastern region correlates with lower COVID incidences. However, it may also reflect GPs’ hesitancy to further invest, e.g., in certified video consultation set-ups, as practices are privately owned in Germany.

### Comparison with international data

The observed interest in applying most COVID-19 recommendations in non-pandemic times was also exceptionally high among German GPs, surpassing adherence rates from other settings during the pandemic. A cross-national survey conducted among 4,466 GP practices from 37 European countries and Israel revealed increased adherence to infection prevention and control (IPC) measures during the COVID-19 pandemic. Specific improvements were observed in the proportion of staff members who never wore nail polish (rising from 34 to 46.2%) and who never wore rings/bracelets (rising from 16.1 to 32.3%), and in practices implementing a cleaning protocol (increasing from 54.9 to 72.7%) [6]. A cross-sectional study involving 187 Israeli physicians reported that 73% complied with the hygiene rules proposed by the Israeli Ministry of Health (MOH) [32].

Considering the low to moderate levels of COVID-19 incidence and related deaths in Germany, the observed high levels of interest toward the future application of organisational changes among German GPs in this study can be explained using the Health Belief Model [31]. German GPs may have perceived COVID-19 as dangerous and influenced their preventive behaviour, which is in line with the Health Belief Model (HBM), suggesting a connection between individuals’ perception of disease severity and their perceived susceptibility to it in influencing preventive health behaviour. Perceived severity encompasses beliefs about the negative consequences of contracting an infection, while perceived susceptibility pertains to beliefs about one’s vulnerability to an infection. As a result, German GPs may have committed more strongly to the COVID-19 hygiene recommendations. A cross-sectional study for the German region of Hamburg explored the properties of the HBM as predictors of the public’s compliance with COVID-19 preventive behaviour [36]. Cues to action and perceived benefits positively influenced vaccination uptake in Hamburg adults, while face mask adherence was similarly influenced by perceived susceptibility, seriousness, and benefits, but perceived barriers negatively impacted both. These results suggest that public health professionals should consider individuals’ health beliefs when designing effective COVID-19 prevention programs. Future public health messaging should emphasise the benefits of precautionary actions and the potential lethality of COVID-19 to raise individual concerns appropriately.

Furthermore, GPs’ preventive behaviour and adherence to hygiene rules during the pandemic may have contributed to Germany’s general country-wide policy of COVID-19 containment. The successful adoption among German GPs may have contributed to the low COVID-19 incidences. Our findings support this explanation that most organisational practice changes (7 out of 11) were rated positively by German GPs.

### Important predictors for applying organisational changes in non-pandemic times

The identified critical factors associated with a lower interest in the future use of the DEGAM COVID-19 guideline in non-pandemic times were being male, having extensive GP practice experience (28 or more years), treating fewer than 1,000 patients per month in the practice, practising in Eastern Germany, not using the Corona-Warn-App, and having lower crisis leadership skills. Due to the lack of research conducted in GP settings, comparing these findings with research conducted in general populations is only possible. These studies found, mainly in line with our findings, that characteristics related to increased health knowledge and public awareness are associated with COVID-19-related preventive behaviours:

A review including 29 studies on adherence to COVID-19 public health guidelines in Western countries identified significant factors associated with higher adherence to COVID-19 public health guidelines, e.g., female gender, perceive COVID-19 as threatening, and usage of traditional news media [22]. A systematic review of strategies to improve adherence to the preventive measures against COVID-19 using 18 electronic databases revealed further critical factors associated with favourable attitudes and behaviour of people, which promoted adherence to COVID-19 prevention [10]. These factors covered increased health knowledge, transparent communication, and public awareness about COVID-19 risks and preventive measures. A cross-sectional study among 20,947 adults of the general population in the United Kingdom reported that lower self-reported adherence to COVID-19 preventive behaviours was related to young age, high risk-taking behaviour, low confidence in government, and low empathy.

### Regional differences

The observed lower interest in the future application of organisational changes in Eastern Germany could be explained by past lower public awareness towards COVID-19 due to lower incidences in this region. GPs in Eastern Germany could have perceived COVID-19 thus as less dangerous than their counterparts in other regions. However, the patient population in Eastern Germany was older [7], which objectively faces a higher risk of COVID-19-related complications and deaths.

GPs’ practice experience – a proxy for age – was found to be related to the practical use of the organisational changes (Supplementary Table S2) and the future use of the DEGAM COVID-19 guideline. However, age differences between GPs in Eastern Germany compared to the other regions as a possible explanation can be excluded, at least for the future use of the guideline, as the multivariate model was controlled for the years of practice experience.

Another interesting finding was the positive association between better crisis leadership skills and the interest in the future use of the DEGAM COVID-19 guideline in non-pandemic times among GPs from Western, Northern, and Eastern Germany, but not among GPs from Southern Germany. Additional analyses revealed that this association was even significant in the bivariate model for Southern Germany, suggesting that it was not explained by differences in the composition of GPs or their practices. Moreover, this was not attributable to limited variance in leadership skills among GPs from Southern Germany. One explanation could be that leadership skills are only relevant in regions with lower levels of interest in the implemention of the DEGAM guideline when applying organisational changes. GPs’ from Southern Germany showed indeed the highest interest. However, more research using qualitative approaches is needed for a detailed understanding.

### Strengths and limitations

The data collection process was based on a multi-level clustered randomised selection from all existing general practitioners (GPs) in Germany. By including regional identifiers in the dataset, we were able to explore spatial variations. Including ratings for individual organisational practice changes made it possible to compare the overall interest and the interest in applying specific changes. This study went beyond assessing past adherence to the guideline by considering its application in non-pandemic times, which is thus essential for creating pandemic preparedness concepts in primary care. The extensive range of explored variables helped to examine individual and practice-level factors. The NPS used in this study allows for benchmarking of included organisational changes and enables valid comparisons with future research using the NPS.

Data collection occurred earlier in the pandemic, so the interest in applying the DEGAM COVID-19 guideline may have changed over time. The factors influencing GPs’ interest in implementing recommendations in non-pandemic times should be interpreted in the context of working under pandemic conditions. This study focused solely on GPs, neglecting other healthcare providers in the German primary care sector. The response rate from GPs for the survey was likely low due to the time-consuming vaccination campaign. Thus, a selection bias may have influenced the findings despite the sample being based on randomisation. However, this study still encompassed a large number of general practitioners across different regions in Germany.

### Advancing future (regional) pandemic preparedness in general practice

Future pandemic preparedness in GP practices should incorporate the experiences from the COVID-19 pandemic [17, 26, 28], such as:

First, a significant percentage of people have underlying risk factors, e.g., smoking, obesity, or cardiovascular diseases, that could lead to severe infections [25]. Therefore, GPs should adhere to the guidelines when treating such vulnerable subpopulations. Second, future guidelines to prevent infectious respiratory diseases may consider mandating FFP/(K)N95 masks, which are highly effective in protecting against COVID-19 infections. However, it is essential to note that frequent coughing has been reported as a side effect [23]. Third, many people perceived COVID-19 self-testing as unreliable, partly due to concerns about proper compliance with execution instructions. Therefore, standardised testing conducted by trained staff may be preferred in future pandemic situations to protect medical staff and patients better [24].

In addition, further cross-country studies are necessary [8] to investigate reasons why the interest in applying the COVID-19 guideline in non-pandemic times is high in German GPs. Drawing on the concept of regional pandemic preparedness [9], future studies should focus on smaller spatial units such as municipalities or zip-code areas to regard also subtle regional variations. Qualitative approaches should complement this research, which is valuable for uncovering underlying reasons for the observed regional differences. By considering a nationwide perspective and spatial nuances, pandemic preparedness efforts can benefit from more holistic implementation strategies addressing regional peculiarities.

## Conclusions

The commendable interest of GPs in adhering to the DEGAM COVID-19 guideline during non-pandemic periods positions them as crucial pillars for pandemic preparedness and coping with future crisis scenarios in Germany. Implementing leadership training programs can effectively enhance GPs’ readiness to contribute to pandemic preparedness. Adopting a region-specific approach in conjunction with a nationwide strategy is crucial to ensure preparedness for future pandemics.

## Supplementary Information

Supplementary Material 1.

## Data Availability

The datasets generated and/or analysed during the current study are available from the corresponding author on reasonable request.
