# Supplementary material for: Higher interest to continue COVID-19 practice recommendations in non-pandemic times among German GPs with better crisis leadership skills (egePan study)
Source: BMC Health Serv Res. 2024 Nov 13;24:1396. doi: 10.1186/s12913-024-11855-7 (PMC11562362; doi:10.1186/s12913-024-11855-7)
Supplement: Supplementary file 1 — Supplementary Material 1. [file 12913_2024_11855_MOESM1_ESM.docx]

**Supplemental Material**

**Higher interest to continue COVID-19 practice recommendations in non-pandemic times among German GPs with better crisis leadership skills (egePan study)**

*Benjamin Aretz, Yelda Krumpholtz, Simon Kugai, Nicola Amarell, Manuela Schmidt, Birgitta Weltermann*

**Supplementary Table S1:** GPs’ interest to apply the organizational practice recommendations in non-pandemic times, mean (SD)

**Supplementary Table S2.** Net-Promoter-Scores on the GPs’ interest to apply the organizational practice recommendations in non-pandemic times stratified by GPs’ practice experience

*This supplemental material has been provided by the authors to give readers additional information about their work.*

**Supplementary Table S1:** GPs’ interest to apply the organizational practice recommendations in non-pandemic times, mean (SD)

|  | **Regional level** | | | | | **ANOVA** |
| --- | --- | --- | --- | --- | --- | --- |
| **Structure/Process** | Total sample  (n = 516) | GPs from Western  Germany  (n = 185) | GPs from Northern  Germany  (n = 82) | GPs from Eastern  Germany  (n = 108) | GPs from Southern Germany  (n = 141) | F-statistics (p-value) |
| Disinfectant dispensers at entrance | 8.55  (2.70) | 8.02  (2.28) | 8.50  (2.72) | 7.46  (3.47) | 8.78  (2.29) | **8.41 (< 0.001)** |
| Optimized consultation scheduling to reduce waiting times | 8.00  (2.63) | 8.24  (2.48) | 7.72  (2.66) | 7.40  (3.00) | 8.29  (2.45) | **3.34 (0.019)** |
| Glass screens at reception | 7.03  (3.58) | 7.22  (3.51) | 7.34  (3.14) | 6.77  (3.43) | 6.79  (3.76) | 0.79 (0.499) |
| Face masks obligatory for patients with respiratory infections | 6.96  (3.19) | 6.81  (3.29) | 7.32  (2.81) | 6.83  (3.30) | 7.04  (3.19) | 0.57 (0.632) |
| Phone consultations for patients with respiratory infections | 6.94  (3.12) | 7.11  (2.97) | 6.63  (3.16) | 6.31  (3.63) | 7.36  (2.78) | **2.79 (0.040)** |
| Separate consultations for infectious patients | 6.84  (3.65) | 6.71  (3.43) | 7.10  (3.28) | 6.86  (7.83) | 6.84  (3.40) | 0.25 (0.860) |
| Testing for COVID only if personal protective equipment is sufficient | 6.55  (3.65) | 6.46  (3.66) | 6.83  (3.44) | 6.22  (3.83) | 6.75  (3.64) | 0.62 (0.602) |
| Prescriptions by mail | 6.40  (3.29) | 6.69  (3.23) | 6.04  (3.08) | 6.35  (3.53) | 6.26  (3.30) | 0.91 (0.436) |
| Insurance card reader handled by patients | 5.08  (4.29) | 5.18  (4.10) | 5.61  (4.31) | 3.23  (4.08) | 6.06  (4.27) | **10.09 (<0.001)** |
| Only selected staff treats infectious patients | 4.85  (3.83) | 4.63  (3.77) | 5.07  (3.75) | 4.49  (3.94) | 5.28  (3.85) | 1.22 (0.300) |
| Video consultations for patients with infections | 3.26  (3.52) | 3.56  (3.57) | 2.89  (3.26) | 2.90  (3.44) | 3.36  (3.66) | 1.17 (0.320) |

* Bold values denote significant results with p < 0.05.

**Supplementary Table S2:** Net-Promoter-Scores on the GPs’ interest to apply the organizational practice recommendations in non-pandemic times stratified by GPs’ practice experience

|  |  | **GPs’ practice experience** | | | |  |
| --- | --- | --- | --- | --- | --- | --- |
| **Structure/ process** | **Total sample**  **(n = 516)** | **0-11 years**  (n = 143) | **12-18 years**  (n = 125) | **19-27 years**  (n = 129) | **>27 years**  (n = 119) | **ANOVA** F-statistics (p-value) |
| Disinfectant dispensers at entrance | 64.8 | 75.2 | 65.1 | 60.3 | 56.4 | 1.58 (0.192) |
| Optimized consultation scheduling to reduce waiting times | 44.3 | 52.7 | 55.8 | 46.2 | 17.1 | **3.36 (0.019)** |
| Glass screens at reception | 22.4 | 23.5 | 38.6 | 15.9 | 10.3 | 1.26 (0.287) |
| Separate consultations for infectious patients | 9.9 | 24.1 | 23.7 | 5.0 | -17.8 | 2.36 (0.071) |
| Face masks obligatory for patients with respiratory infections | 9.6 | 25.9 | 23.9 | -1.0 | -14.3 | 2.46 (0.062) |
| Testing for COVID only if personal protective equipment is sufficient | 2.7 | 4.7 | 11.1 | 11.0 | -17.7 | 2.06 (0.105) |
| Phone consultations for patients with respiratory infections | 4.5 | 25.8 | 13.6 | -9.1 | -12.4 | **3.07 (0.027)** |
| Prescriptions by mail | -15.4 | -12.3 | -18.3 | -14.9 | -16.8 | 0.10 (0.958) |
| Insurance card reader handled by patients | -20.1 | 2.3 | -17.1 | -25.7 | -43.6 | **5.57 (0.001)** |
| Only selected staff treats infectious patients | -42.3 | -33.3 | -39.8 | -38.6 | -59.2 | 1.99 (0.115) |
| Video consultations for patients with infections | -72.5 | -74.4 | -70.4 | -64.9 | -81.5 | **3.10 (0.026)** |

* Bold values denote significant results with p < 0.05.
